# Transfer Learning for the Detection and Diagnosis of Types of Pneumonia including Pneumonia Induced by COVID-19 from Chest X-ray Images

**DOI:** 10.3390/diagnostics11081480

**Published:** 2021-08-16

**Authors:** Yusuf Brima, Marcellin Atemkeng, Stive Tankio Djiokap, Jaures Ebiele, Franklin Tchakounté

**Affiliations:** 1 African Institute for Mathematical Sciences (AIMS), Kigali P.O. Box 7150, Rwanda; jaures.ebiele@aims.ac.rw; 2 Department of Mathematics, Rhodes University, Grahamstown 6140, South Africa; 3 Department of Arts, Technology and Heritage, Institute of Fine Arts, University of Dschang, Foumban P.O. Box 31, Cameroon; stive.djiokap@aims.ac.rw; 4 Department of Mathematics and Computer Science, Faculty of Science, University of Ngaoundéré, Ngaoundéré P.O. Box 454, Cameroon; tchafros@gmail.com

**Keywords:** COVID-19, X-ray image, deep transfer learning, diagnosis

## Abstract

Accurate early diagnosis of COVID-19 viral pneumonia, primarily in asymptomatic people, is essential to reduce the spread of the disease, the burden on healthcare capacity, and the overall death rate. It is essential to design affordable and accessible solutions to distinguish pneumonia caused by COVID-19 from other types of pneumonia. In this work, we propose a reliable approach based on deep transfer learning that requires few computations and converges faster. Experimental results demonstrate that our proposed framework for transfer learning is a potential and effective approach to detect and diagnose types of pneumonia from chest X-ray images with a test accuracy of 94.0%.

## 1. Introduction

COVID-19 was declared to be the most lethal pandemic the world has had to grapple with with in recent human history [1,2,3]. Its origin of the first transmission remains unknown; the first reported cases were in December 2019 in Wuhan and has lead to massive loss of life and stagnation of the global economy. The primary mode for the virus transmission from an infected person’s mouth or nostrils in small fluid particles is through coughing, sneezing, speaking, singing, or breathing heavily. The fluid particles vary significantly in size, ranging from larger respiratory droplets to smaller aerosols. They cannot spread person-to-person without coming in close contact because the droplets are too heavy to travel more than a meter. It has been indicated that COVID-19 can be in the air for up to 3 h, 4 h on copper surfaces and nearly 72 h on plastic and stainless materials [3]. Nonetheless, identifying the exact nature of the virus remains an open problem in the medical research community. Early and accurate detection of this viral pneumonia in asymptomatic cases is vital to reducing the transmissibility of the viral infection, the burden on healthcare capacity and the overall mortality rate. Machine Learning (ML) is increasingly being integrated into healthcare systems ranging from medical image acquisition to reconstruction, outcome analytics, and prediction. Thus, the use of ML to detect and classify traditional pneumonias from the pneumonia induced by COVID-19 is vital to providing an early, fast and efficient diagnosing mechanism [4]. Although mass vaccination campaigns are being carried out worldwide, coronavirus cases have been rising largely due to emerging variants of the viral disease. The global death toll of COVID-19 has been markedly increasing. India has surpassed Brazil as the second country with the highest infections and is currently experiencing the third wave of infection and COVID-19 related deaths. Virologists around the world have been extensively working to develop COVID-19 vaccines. Several COVID-19 vaccines have been approved for vaccination and are being used in many countries around the world for COVID-19 immunization. For example, candidates such as Pfizer-BioNTech is an mRNA-based COVID-19 vaccine developed in Germany by the biotechnology company BioNTech in collaboration with the American company Pfizer, Moderna is also an mRNA-based coronavirus disease vaccine developed by Moderna, the United States National Institute of Allergy and Infectious Diseases (NIAID) in association with the Biomedical Advanced Research and Development Authority (BARDA), Oxford-AstraZeneca is amongst the class of viral vector vaccines for prevention and was jointly developed in the United Kingdom by Oxford University and AstraZeneca, CoronaVac (Sinovac COVID-19 vaccine) is an inactivated virus coronavirus vaccine developed by Sinovac Biotech a company in China, Sputnik V (Gam-COVID-Vac) is an adenovirus viral vector vaccine for coronavirus disease developed by the Gamaleya Research Institute of Epidemiology and Microbiology in Russia. Large-scale clinical trials on the safety and efficacy of these vaccines have indicated their effectiveness with minor side effects on tested demographics [5]. Clinical trials of Sinovac in Brazil, Chile, Indonesia, the Philippines, and Turkey have shown 67% effectiveness against symptoms while reducing hospitalisations, intensive care visits and deaths by 85%,89%, and 80%, respectively [6]. The vaccine was developed using conventional technology similar to BBIBP-CorV and BBV152; other inactivated-virus COVID-19 vaccines. The primary advantage of Sinovac is that it does not need to be frozen as does Moderna’s vaccine, which needs to be stored at −20 °C. The AstraZeneca vaccine requires regular fridge temperature and Pfizer’s vaccine at −70 °C [5,7]. Secondly, both the vaccine and raw material for formulating the new doses could be transported and refrigerated at 2–8 °C temperatures, at which flu vaccines are also kept. This compounded advantage makes the vaccine suitable for developing countries that have limited public health infrastructure. Sputnik V was developed by Gamaleya Institute in Russia and is currently being used in Belarus, United Arab Emirates, Saudi Arabia, India, and Iran [8]. However, mass vaccine adaption in many countries remains a public health logistical (e.g., logistics of manufacturing, storing and distributing the vaccine, and mass vaccination) and leadership challenge. The problem is attributed to many reasons ranging from citizens’ vaccine resistance to vaccine nationalism [9,10,11]. With the development and usage of vaccines, further research is needed to address open questions such as: will the new vaccines be able to control the COVID-19 pandemic? What is the efficacy of current vaccines on the emerging variants of COVID-19 from the UK, South Africa, Brazil, Portugal, and India identified as more contagious and lethal? What will be the long-term efficacy and side effects of current vaccines that have been researched, developed and tried at break-neck speed on different population demographics? Giving the upsurge in vaccine nationalism, will it be possible to surmount both financial and political challenges for equitable distribution of vaccines, especially to low-and-middle-income countries?

### 1.1. Problem Statement

One of the critical steps in containing viral spread is the timely detection of positive cases in the community. Clinical laboratories have been developing, validating, and implementing various molecular and serologic assays to test SARS-CoV-2 nucleic acid [12]. Reverse Transcriptase-Polymerase Chain Reaction (RT–PCR) is a laboratory testing method that combines reverse transcription of RNA into DNA (called complementary DNA or cDNA) and amplification of specific DNA targets using the standard Polymerase Chain Reaction (PCR). RT–PCR diagnostic has been identified to be effective in detecting the SARS-CoV-2 virus. This technique, however, has inherent limitations such as long delays in obtaining test results, patients with high clinical suspicion testing falsely negative on initial RT-PCR test often requiring multiple tests runs to validate the result and a slew of other laboratory logistical challenges [12,13]. Low test sensitivity may be possible due to: sub-optimal clinical sampling approaches; variations in viral load; and manufacturer test kit sensitivity. With communities having a high surge in caseloads, managing these RT-PCR negative patients are overwhelmingly cumbersome. Procedural adherence requirements in the laboratory and a multitude of the testing characteristics could be attributed to the limitations [12,13,14,15]. Laboratories and virology research centres are working towards overcoming the current limitations of RT-PCR testing in enabling more accurate detection of the coronavirus. According to the World Health Organization recommendations of October 2020, chest imaging examination is a useful and effective approach for detecting clinical symptoms of COVID-19 suspected and recovered cases [16,17]. These imaging modalities include ultrasound, X-rays, MRI of the chest, computed tomography (CT) and needle biopsy of the lung. Among these modalities, the chest X-ray is primarily used to detect coronavirus in contrast to CT, MRI and other medical imaging modalities. The CT image takes longer for imaging, and CT scanners are sparsely available in low-income countries. Additionally, CT imaging is costly, and it may pose health risks to pregnant women and children due to its high ionizing radiations [18]. In stark contrast, X-ray imaging has a multiplicity of use cases in many medical and epidemiological applications because it is readily available around the world [19,20]. Thus, the chest X-ray is a well-suited modality for examining and diagnosing cases due to its lightweight operating speed, lower cost and ease of use by radiologists. However, prior research has indicated some degree of inconsistencies in chest X-ray images of COVID-19 patients [21].

### 1.2. Objectives

This work aims to detect and classify three types of pneumonia (lung opacity pneumonia, COVID-19 pneumonia, and viral pneumonia) and distinguish these types of pneumonia from a healthy chest X-ray scan to aid safe, accurate, less cumbersome and timely diagnosis. We aim to use domain-invariant representations from a source domain to transfer unto the chest X-ray target domain to improve model prediction performance given limited target domain sample data without overfitting. Moreover, we provide a framework of end-to-end learning using a dataset collected from multiple locations and periods to study the transferrable properties of latent representations across domains and tasks using transfer learning.

### 1.3. Contributions

Our main contribution in this work is a novel end-to-end Deep Transfer Learning framework using deep convolutional neural network that detects and classifies three types of pneumonia from chest X-ray scans. This study used the public COVID-19 Radiography dataset collected from more than 20 hospitals across the world [22]. In this dataset, we have a total of 21,165 chest X-ray images in which 3616 images are infected by pneumonia induced by COVID-19, 6012 images are lung opacity infection, 1345 images are viral pneumonia, and 10,192 images are normal images that are not infected. We review the most recent 16 papers applying ML to classify the different types of pneumonias from chest X-ray images. We found that most published ML models are dealing with limited data that are mostly two classes (COVID-19 pneumonia and uninfected chest X-ray) or three classes (COVID-19 pneumonia, all the other types of pneumonia regrouped into a single class, and uninfected chest X-ray). Among the 16 most recent papers reviewed, we found two papers dealing with four classes and both resulting in an accuracy less than 90%. These two works are based on limited data i.e., one used 1251 X-ray images and another 5941 X-ray images. We proposed a ResNet50 CNN architecture that is built to detect and classify four types of classes (lung opacity infection, viral pneumonia, pneumonia induced by COVID-19, uninfected chest X-ray) with an accuracy of 94.0% using 21,165 chest X-ray images with a well-adopted methodology to deal with class imbalance. The ResNet50 performance in convergence and generalization is in contrast to Alexnet, VGG, and ResNet34.

### 1.4. Outline

This paper is organized as follows: In Section 2, the reader is introduces to the problem background and related literature, in Section 3, the research methodology is described. In Section 4, experimental results are presented and a comparative survey of performance of existing literature is given. Section 5 discusses the result of the paper, and conclusions and future research directions are outlined in Section 6.

## 2. Related Literature

Machine learning techniques have been used extensively in health informatics, from drug discovery to epidemiological modeling and diagnosis of deseases from medical images [23,24,25]. The use of machine learning in healthcare analysis has been enhanced by the increased availability of datasets to the research community and advancements in architectures for modeling scientific events. The outbreak of the COVID-19 pandemic has seen machine learning, deep learning and hybrid methods used to model the non-linear and complex nature of the spread of the SARS-CoV-2 virus with a capacity of higher generalization and predictive reliability in longer time windows [26,27,28,29].

There has been an extensive body of scholarly work to detect COVID-19 from chest X-ray (XCR) and CT images. These methods are varied in their use of different pipelines and ML techniques from feature preprocessing to the choice of architecture under different contexts and considerations, thus yielding different performance results. Ahammed et al. [30] in their comparative survey of ML and deep learning approaches for the detection of COVID-19 using a dataset of all publicly available chest X-ray images of COVID-19 patients, reported 94.03% accuracy, 95.52% AUC, and 94.03% sensitivity. Ng et al. [21] created a massive dataset of 13,1975 XCR images and used a deep neural network model to classify the images which resulted to an accuracy of 93.30%. Abbas et al. [31] developed a convolutional neural network (CNN) model named Decompose-Transfer and Compose (DeTraC) to classify chest X-ray images where they reported accuracy of 93.1% and 100% sensitivity. In their study, Apostolopoulos et al. [32] using a dataset of 1,427 XCR images and a transfer learning approach, reported the accuracy of 96.78%, a sensitivity of 98.66% and specificity of 96.46%.

El-Din Hemdan et al. [33] proposed a Computer-Aided Diagnosis system (COVIDX-Net) to classify positive and negative COVID-19 cases with reported F1-Scores of 89% and 91% for normal and COVID-19, respectively. Karar et al. [34], developed a cascaded architecture of VGG-16, ResNet-50V2 and DensNet-169 which achieved the accuracy of 99.9%. Minaee et al. [35] used radiograms of 5000 chest X-ray images to perform transfer learning using ResNet-18, ResNet-50, SqueezeNet and DenseNet-121 with a reported best sensitivity of 98% and specificity of 90%. Heidari et al. [36] used histogram equalization and bilateral filtering techniques for preprocessing resulting in filtered images. These features were used to train a VGG-16 network obtaining the best accuracy of 94.5% on all image classes and 98.1% accuracy on COVID-19. Khan et al. [37] proposed CoroNet based on the Inception architecture. They used the model to classify Normal, Pneumonia-bacterial, Pneumonia-viral and COVID-19 from chest X-ray images. Their model achieved an overall accuracy of 89.6% with 93% precision and 98.2% recall for the COVID-19 class. In their work, Chandra et al. [38] proposed an Automatic COVID-19 Screening (ACoS) system using a two-staged majority voting scheme of an ensemble of models. They reported a validation accuracy of 98.062% in the first stage and 91.329% accuracy in the second stage. Ismael et al. [39] used a pre-trained ResNet-18, ResNet-50, ResNet-101, VGG-16 and VGG-19 models for feature extraction from XCR images. Using a Support Vector Machine (SVM) classifier applied with different kernels, they obtained the accuracy of 94.7% on ResNet and SVM with a linear kernel.

Karthik et al. [40] developed a custom CNN model which learns latent feature filters. Their model has a reported F1-Score of 97.20% and an accuracy of 99.80%. Ohata et al. [41] used a pre-trained MobileNet model with a linear SVM classifier and DenseNet-201 with a Multi-Layer Perceptron to detect COVID-19. They reported accuracy and F1-Score of 95.6% for the MobileNet model and 95.6% accuracy and F1-Score for the DenseNet-201 model. De Moura et al. [42] demonstrated three end-to-end models for the classification of chest X-ray images from portable equipment using a dataset of 1,616 images. Their proposed DenseNet-201 CNN models have a reported accuracy of 79.62%, 90.62%, and 79.86%, respectively. Duran-Lopez et al. [43] proposed a deep learning model (COVID-XNet) trained using five-fold cross-validation. They obtained an accuracy of 94.43% and an AUC of 98.8%. Shorfuzzaman et al. [44] used a pre-trained Convnet encoder with a contrastive loss function to learn the representation of XCR image features. Afterward, the learned features were classified using a Siamese neural network with a reported accuracy of 95.6% and an AUC of 97%. Shankar et al. [45], introduced a hand-crafted feature extraction method (FM-HCF-DLF) and a CNN based on the Inception-V3 architecture for the classification of XCR images. Their model yielded a sensitivity of 93.61%, specificity of 94.56%, accuracy of 94.08%, precision of 94.85%, F1-Score of 93.2%, and Kappa value of 93.5%.

The 16 most recent papers using ML models reviewed above are compared in Table 1 in terms of the size of the total number of images used, number of classes, adopted method, and reported accuracy. Table 1 shows important results or observations; so it warrants a detailed explanation. Out of the 16 papers reviewed, four papers addressed a binary classification with images ranging from 50 to 1531 and reported accuracy ranging from 95% to 99.0%; the average total of images and accuracy in these four papers are 725 images and 96.81% respectively. We have 10 papers that built the ML model based on a dataset with three classes. The reported accuracy ranges from 87% to 99.7% and the data size ranges from 542 to 16,995 images; the average total number of data size and accuracy reported in these 10 papers are 4623 and 94.417%, respectively. Only two papers attempted the work with a dataset of four classes. Of these two, the first published paper reported accuracy of 89.0% using 1251 images while the second paper reported accuracy of 89.92% with 5941 images. From this survey, it can be noted that a few classes classification problems are prompt to have greater accuracy with a small dataset. The accuracy measured on a small amount of data are generally unreliable since the model can be generalizing very poorly. In addition, most of the datasets discussed are not balanced with the number of COVID-19 cases ranging from 25 images to 1968 images. Some of the papers did not report the number of images per class. We reported an accuracy of 94.0% using a larger data that includes 3616 images of COVID-19, thus guarantees generalization compared to the above four classes classification papers.

## 3. Methods

This section provides discussion on the transfer learning framework and the related deep transfer learning setting. We elucidate the conceptual framework of transfer learning in medical image analysis. Also, an exploratory data analysis is carried out to understand the dataset and its inherent characterization. In addition to this, we state the performance criteria for the proposed framework for generalization.

### 3.1. Transfer Learning

We provide the formal notation for transfer learning. Consider the source domain $D_{S}$ as:

$$D_{S}=\left\{X_{S},\;P\left(X_{S}\right)\right\},$$

where $X_{S}$ is the input space and $P(X_{S})$ is the marginal probability of the input. Te source input $X_{S}\subset X_{S}$ is defined as:

$$X_{S}={\left\{x_{S_{i}}|\forall x_{S_{i}}\in R^{n}\right\}}_{i=1}^{m},$$

where *m* is the number of vectors $x_{S_{i}}$ of size *n*. In $D_{S}$, a source task $T_{S}$ is defined as:

$$T_{S}=\left\{Y_{S},\;P\left(Y_{S}|X_{S}\right)\right\},$$

where $Y_{S}$ is the label space and $P(Y_{S}|X_{S})$ is the conditional probability of the output given the input. This suggests that if $Y_{S}\subset Y_{S}$ then the source output is:

$$Y_{S}=\left\{y_{S_{1}},y_{S_{2}},\ldots ,y_{S_{m}}\right\},\;\forall y_{S_{i}}\in Y_{S}.$$

Each of the $y_{S_{i}}\in \left\{c_{1},c_{2},\ldots ,c_{k}\right\}$ with $c_{k}$ a given class. A target domain $D_{T}$ is defined as:

$$D_{T}=\left\{X_{T},P\left(X_{T}\right)\right\},$$

where $X_{T}\;\mathrm{is}\;\mathrm{the}\;\mathrm{input}\;\mathrm{space}\;\mathrm{and}\;P\left(X_{T}\right)\;\mathrm{is}\;\mathrm{the}\;\mathrm{marginal}\;\mathrm{probability}\;\mathrm{of}\;\mathrm{the}\;\mathrm{input}.$ The target input $X_{T}\subset X_{T}$ is given by:

$$X_{T}={\left\{x_{T_{i}}|\forall x_{T_{i}}\in R^{n}\right\}}_{i=1}^{m}.$$

For source target domain $S_{T}$, a target task $T_{T}$ is defined as:

$$T_{T}=\left\{Y_{S},P\left(Y_{T}|X_{T}\right)\right\},$$

where $Y_{T}$ is the label space, $P(Y_{T}|X_{T})$ is the conditional probability of the output given the input. The output, $Y_{T}\subset Y_{T}$ is defined as:

$$Y_{T}=\left\{y_{T_{1}},y_{T_{2}},y_{T_{3}},\ldots ,y_{T_{m}}\right\},\;\forall y_{T_{i}}\in Y_{T}.$$

The hypopaper space $H\subset Y^{X}$ is defined as

f:X→Ys.t.f∈H

X↦f(X).

The goal of transfer learning is to learn a representation $\tilde{X}$ such that

$$\tilde{X}=\underset{f\in H}{\mathrm{argmin}}\left\{L\left(f\left(X\right)\neq Y_{}\right),\right\}$$

where L is the loss function defined as:

L:Y×Y→R

$$L\left(y,f(X)\right)\mapsto R.$$

The classification empirical risk $R_{D_{T}}$ is measured by:

$$R_{D_{T}}=P\left(f\left(X_{T}\right)\neq y_{T}|\tilde{X}\right),\;D_{S}\neq D_{T}\;\mathrm{or}\;S_{T}\neq T_{T},$$

such that:

$$f_{D_{T}}^{*}=\underset{f\in H}{\mathrm{argmin}}\left\{R_{D_{T}}\left(f\left(X_{T}\right),Y_{T},\tilde{X}\right)\right\}.$$

### 3.2. System Architecture

**Figure 1:**
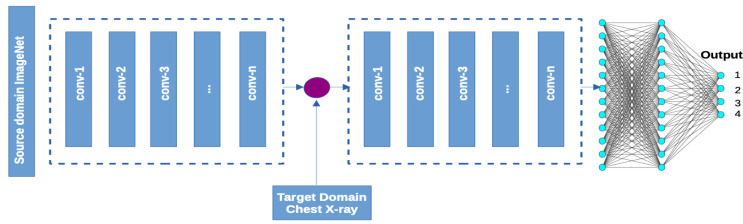
Proposed network architecture where convolution blocks in the left segment indicate pre-trained weights from the source domain which remain frozen and the right indicates the frozen base layers with trainable fully connected layers with a top Softmax layer.

CNNs have increasingly been used in vision tasks such as detection, classification, and segmentation [55,56,57,58,59]. CNNs take a biological inspiration from the visual cortex. The visual cortex is a region of the brain that has cells that are sensitive to visual perception. The adoption of CNNs in ML stems from a research conducted by Hubel and Weisel in 1962 where they demonstrated that some individual neuronal cells in the brain fire or activate only in the presence of certain edges and in specific orientations [60]. A CNN architecture consists of a convolutional, pooling which is used for down-sampling then followed by a non-linear activation and a fully connected layers. The convolutional has a filter that acts as a feature detector and selection. The convolved region is known as the receptive field. The output of a convolution is a feature or activation map which serves as input to deeper layers of the network. The role of the filter (a concatenation of kernels) is to detect low level features such as edges, colors, curves, virtual lines, boundaries, and high level features such orientations, local surfaces and discontinuities as was first proposed by Marr [61]. In this paper, ImageNet (i.e., a dataset with over one million images and a thousand categories of objects) was used as the source domain for the basis of transfer learning to chest X-ray images. Figure 1 shows the proposed architectural framework for this representational transfer learning task. Transfer learning is a well-suited framework for healthcare computer vision tasks where target domain datasets for learning are significantly small, and model generalization is a key consideration.

### 3.3. System Model and Assumptions

**Figure 2:**
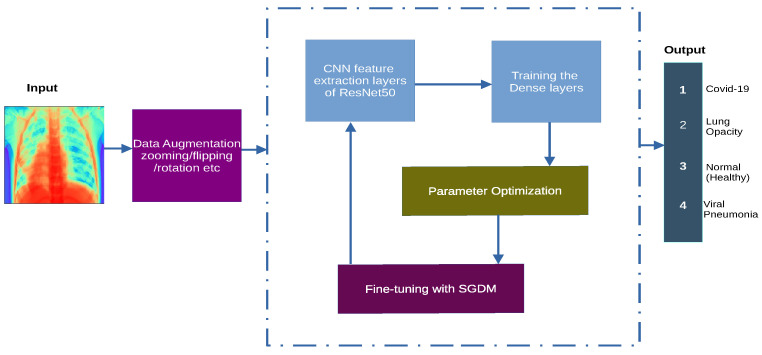
The schematic represents the proposed system model wherein the input is an RGB X-ray tensor, followed by a series of affine transformations. The next stage is a feedback latent feature extraction through convolution operations, classification in the dense layers preceded by non-linearities and parameter optimizations using SDGM. The four output classes are shown in the final stage of the model.

A high-level overview of our proposed transfer learning framework is presented in Figure 2. The initial stage is dataset setup, which includes loading both the brain chest X-ray image scans and associated class labels followed by batch normalization and cross-validation split into train, validation and test sets. We used various data augmentation approaches such as zooming, flipping, rotation, mirroring, etc. to make the model generalize better. Afterwards, we used ResNet50 CNN architecture, Stochastic Gradient Descent for the transfer learning framework.

### 3.4. Dataset

The COVID-19 Radiography Database was used to carry out experiments in this research [22]. This dataset was collected from multiple international sources, at time different timescales [62,63,64,65]. A summary of the dataset is presented in Table 2. All the X-ray images are in Portable Network Graphics (PNG) file format and of 299 × 299 pixel resolution.

**Figure 3:**
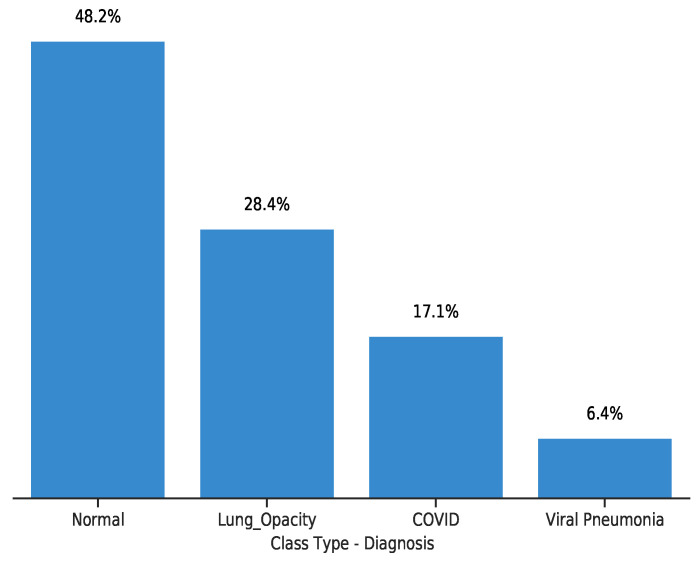
A histogram of the distribution of the X-ray images per class. Healthy/Normal has the largest numbers of data-points, representing 48.2% per cent of the dataset followed by Lung Capacity, COVID-19then viral pneumonia respectively.

To understand the underlying signal distribution in the chest X-ray image dataset, we performed exploratory data analysis. Figure 3 gives a histogram of the dataset class distributions. Healthy and Lung Opacity samples compose 80% of the dataset. Given that our primary objective is to recognise COVID-19 patients to aid early diagnosis thus preemptive medical care, the figure indicates a problem of class imbalance. Viral Pneumonia is the least represented class indicating 6.4% of the total dataset, thus in such situation Precision, F1-Score or Recall are better suited as metrics in contrast to accuracy.

**Figure 4:**
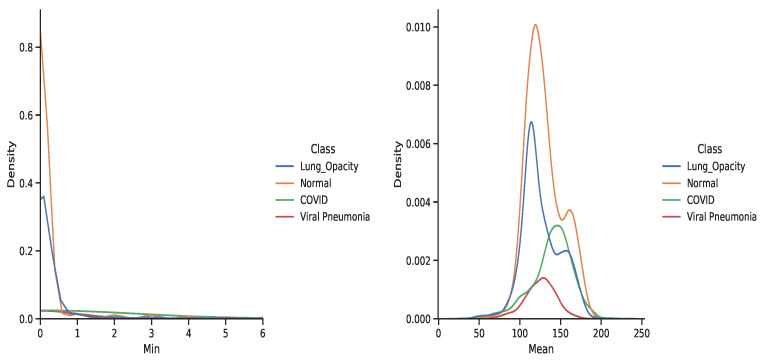
The minimum and mean RGB color intensity distributions for the four X-ray image classes.

**Figure 5:**
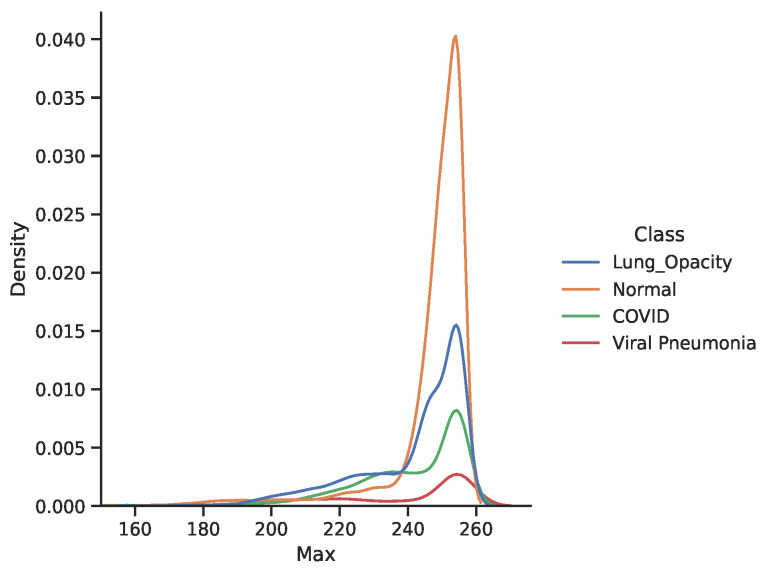
Maximum RGB color intensity distributions for the four X-ray image classes.

The dataset contains a substantial number of images compared to existing literature [4,66,67]. However, due to privacy concerns, additional clinical information about patients is not available. Thus, we proceed with the investigation of image patterns and relationships between the classes. The data are unbalanced with almost 50% of samples belongs to the “Healthy” class that may bias the model towards this class in terms of performance. The X-ray images are rank three tensors that represent the height, width, and number of channels. We proceed by examining the inherent pattern between the image colour values and their class. Figure 4 and Figure 5 show the RGB color intensity distributions for the four classes which is scaled between 0 to 255-pixel intensities for the individual image classes. The distribution illustrates how the minimum, mean, and maximum colour values are presented in the dataset.

We continue by observing the relationship between a sample X-ray image mean as shown in Equation (1) and its standard deviation using Equation (2):

(1) $$\bar{x}=\frac{1}{I_{c}I_{h}I_{w}}\sum_{i}^{I_{c}}\sum_{j}^{I_{h}}\sum_{k}^{I_{w}}x_{ijk},$$

where $I_{c}$ is the number of color channels, $I_{h}$ is the height of the image and $I_{w}$ is the width of the image.

(2) $$\bar{\sigma }=\sqrt{\frac{1}{I_{c}I_{h}I_{w}}\sum_{i}^{I_{c}I_{h}I_{w}}{\left(\sum_{j}^{I_{c}}\sum_{k}^{I_{h}}\sum_{l}^{I_{w}}x_{jkl}-\bar{x}\right)}^{2}}.$$

**Figure 6:**
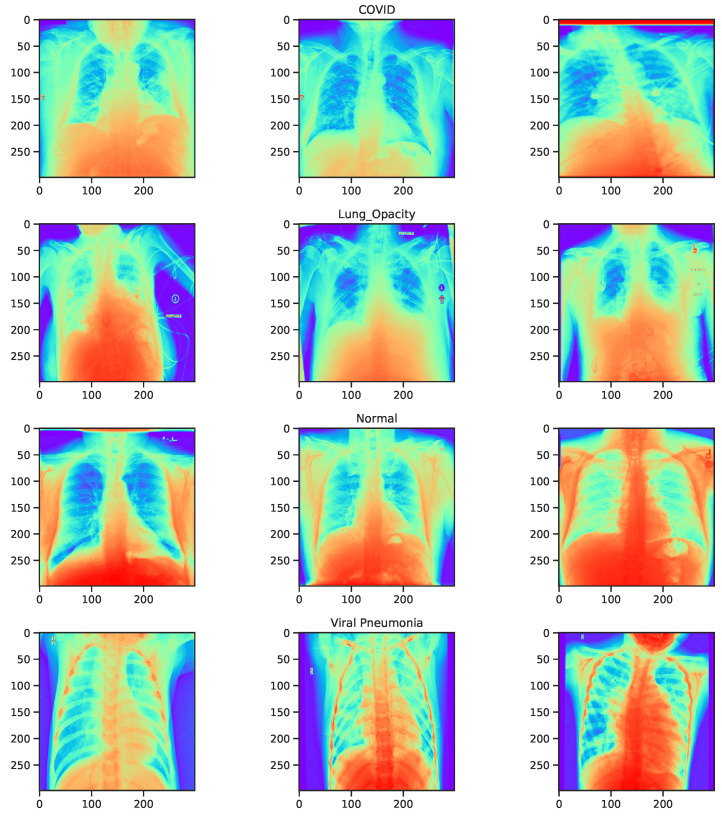
A comparison illustrating a plot of the 3 colors channels.

The distribution of the whole dataset is very akin to the individual healthy and lung opacity patient images, this is because both classes contribute the most to the dataset relative to the remaining classes of viral pneumonia and COVID-19. Separating by class, we can visualise that the mean, maximum, and minimum values vary according to the image class. Amongst all classes, viral pneumonia shows a Gaussian-like distribution across the three different distributions while COVID-19 shows a nearly normal distribution. The maximum value possible for an image is 255 and most classes peak around it. From Figure 4, viral pneumonia is the class that has the highest samples with lower maximum values. Most samples’ RGB colour intensities are within the 200–225 range. Normal (Healthy) and lung opacity images show a very similar distribution of their mean values. This may be due to the two classes having the most sample X-ray images in the dataset. Moreover, the different peaks in the distribution could be attributed to the image source (e.g., two different hospitals where instrument noise, compression error or some other phenomena may have contributed to the veracity of the underlying signals). Figure 4 shows a similar distribution with regards to the maximum values as indicated by the local minimum between intensities of 220 to 240. At the same time, normal patients have a peak at 150 and another peak around 250. We observe that the images are in gray-scale, they have the three channels which contain repeated RGB values. A visualised sample images from the dataset are shown in Figure 6 in a rainbow color map.

**Figure 7:**
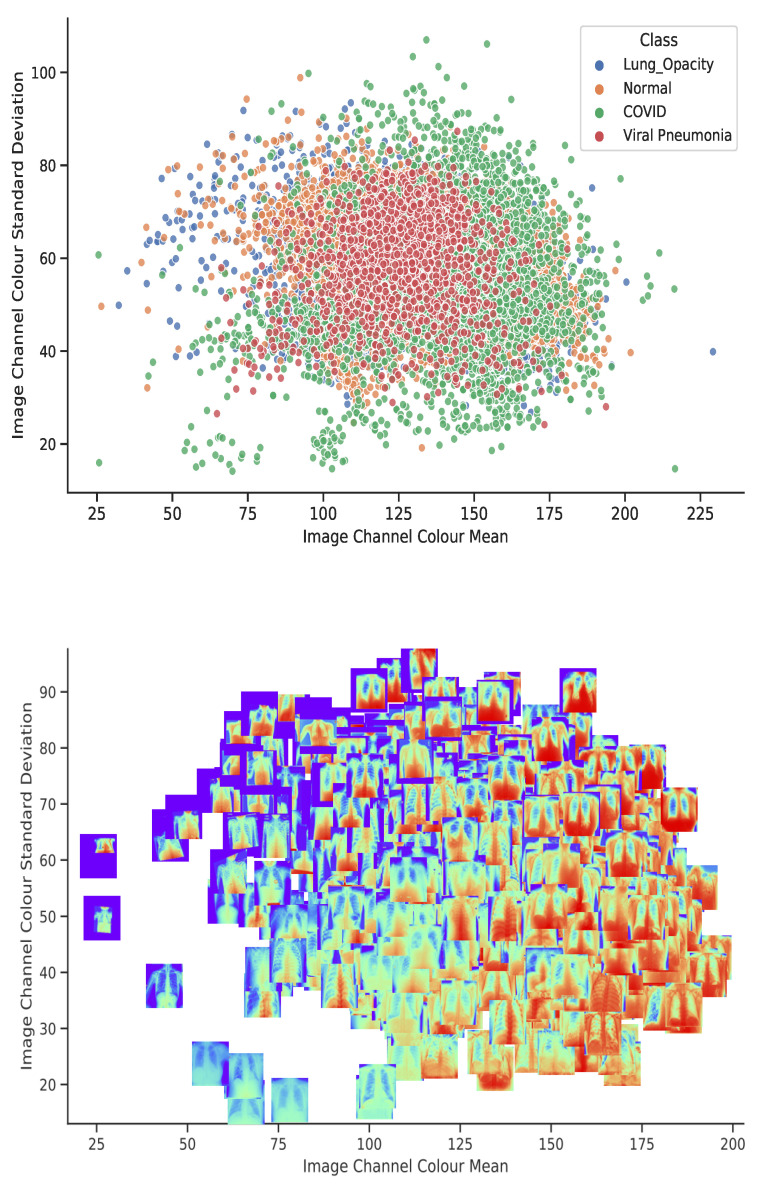
A side-by-side comparison of the dataset clusters using image mean and standard deviation (top-panel) and the 10% (bottom-panel) zoom at the centre of centre mean of the images.

**Figure 8:**
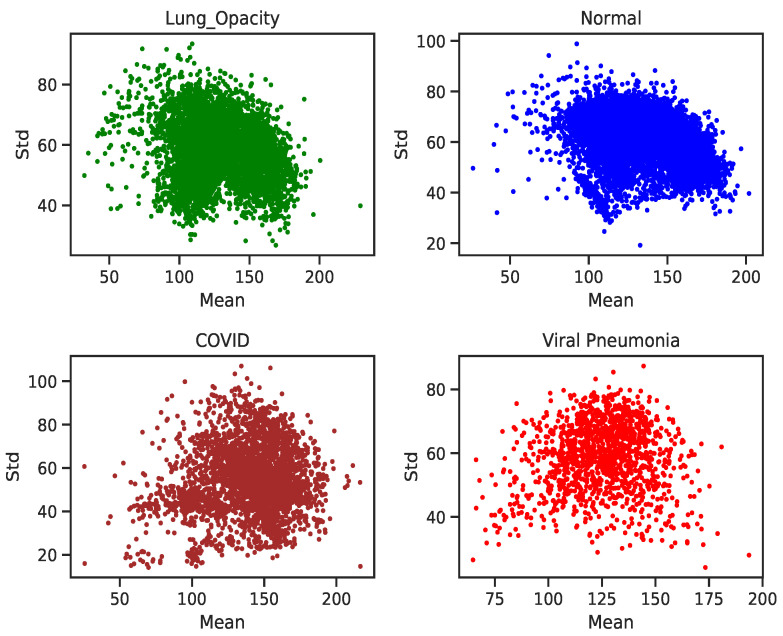
Individual class distributions for COVID-19 (bottom-right) to Healthy (top-left). Normal (healthy) and Lung Opacity images have a similar cluster formation and pixel intensity distribution.

Figure 7, gives a depiction of the dataset projected onto a two-dimensional plane, shows that most of the data points are clustered in the central region of the graph. That implies the inter-class pixel intensity variability is low. However, COVID-19 samples show some clusters with a high channel colour mean and pixel intensities laying between 150 through 200. This can be visualized in Figure 7 (bottom-panel), a 10% zoom in the centre of Figure 7 (top-panel). It can be observed that the samples with a lower mean and a low standard deviation are in the lower corner and the upper corner has the converse situation. Because of the dense clustering of data points in Figure 7, which gives a high-level overview of the dataset but not fine-grain information, a plot of class-level depictions of the dataset is shown in Figure 8. We observe that the classes (COVID-19, Lung Opacity, and Viral Pneumonia) have high intra-class variability with outliers that are distant from the centroid.

Figure 8 shows that Normal (Healthy) and Lung Opacity have similar data point cluster formations. This similarity in distributional pattern between the two classes is characterised by the spread of the respective graphs where a majority of the samples are along regions of high standard deviation, and within 100 to 180 mean pixel intensities. Viral Pneumonia images, on the other hand, show a denser scatter plot which is due to the samples having higher in-class similarity. The graph of the COVID-19 does not indicate any semblance to the other three classes. It has a higher variance and more outliers compared to the other classes. The data points are scattered across all regions of the graph. This phenomenon could indicate the unique intensity distribution of the image signals compared to the other three classes.

### 3.5. Learning Setting

The CNN architectures comprise varied hyperparameter configurations and tuning techniques. Due to the high in-balance nature of the dataset under study, an inverse class-weighting scheme in Equation (3) was used to balance the class weights to help avoid a biased model that performs well on majority class and poor on minority classes:

(3) $$W_{j}=\frac{m}{kn_{j}},$$

where $W_{j}$ is the weight matrix of class *j*, *m* is the total number of training examples, *k* is the total number of classes, and $n_{j}$ is the number of examples belonging to class *j*. Another useful technique that we use to help combat overfitting is the time-based learning rate decaying technique to vary the learning rate over each iteration on the training batch to aid faster convergence as shown in Equation (4):

(4) $$\eta_{t+1}=\frac{\eta_{t}}{1+\rho e_{t}},$$

where $\eta_{t+1}$ is the new learning rate, $\eta_{t}$ is the current learning rate, ρ is the decay rate hyperparameter and $e_{t}$ is the epoch number at time *t*. Another useful, technique to obtain high performance is the use of Stochastic Gradient Descent (SDG) with Momentum. The vanilla SGD is shown in Equation (5), where $\theta_{j}$ is the weight at batch *j* which is updated with respect to the gradient of $\theta_{j}$ that shows the direction of optimization across the loss landscape; η∈[0,1] is the step size, and the loss function L(θ) we seek to minimize.

(5) $$\theta_{j}\leftarrow \theta_{j}-\eta \nabla \theta_{j}L\left(\theta \right).$$

The Stochastic Gradient Descent uses an iterative search approach to find the optimal minimizer (parameters or weights) that minimize the objective function (the loss function) thus obtaining a model that generalizes to OOD examples. However, finding the global minimum (or minima) which is the ideal objective of optimization is a hard problem largely due to saddle points or convergence of the optimization algorithm to a local minima. This optimization challenge is more so common for deep learning problems which are high dimensional and suffer from the curse of dimensionality. Thus Stochastic Gradient Descent with Momentum (SDGM), Equation (6), is a handy optimization technique to overcome the problems of saddle points and local minima.

(6) $$\begin{array}{cc} \; & v_{t+1}\leftarrow \rho v_{t}+\nabla \theta L\left(\theta \right),\\ \; & \theta_{j}\leftarrow \theta_{j}-\eta_{j}v_{t+1}. \end{array}$$

In SDGM, the term *v* is often considered as the velocity and ρ as the frictional force controlling the velocity as the weight parameter θ is updated in each iteration. As a memory unit, the velocity *v*, which has accumulated previous gradient information, address the problem of convergence to saddle points or local minima in the loss landscape which results in SDGM having a better generalization and performance guarantees to SDG.

### 3.6. Simulation Environment

Experiments are carried out on the Google Colaboratory (Colab) platform. Colab provides access to a high performance Virtual Machine (VM) that dynamically allocates NVIDIA K80s, T4s, P4s and P100s Graphic Processing Unit (GPU), Random Access Memory (RAM) and GDrive storage for high-end computing freely.

### 3.7. Performance Metrics

Model performance evaluation is a key constituent in the pipeline of building any ML system. Given that the primary focus of such model is to perform well on unseen future data, therefore, evaluate train, validation and test sets give a good indication on the generalization bounds of such model. In that regard, a confusion matrix is useful metric to help evaluate a classification model. Confusion matrix is an intuitive cross-tabulation of actual class values and predicted class values. It contains a cross-tabulation of every observations that fall in each category.

- Accuracy (acc): a measure that indicates the proportion of correct predictions to the sum of evaluated samples.
  $$\mathrm{acc}=\frac{TP}{TP+FP+TN+FN},$$
  where TP,FP,TN and FN are the True Positive, False Positive, True Negative and False Negative respectively.
- Sensitivity/ Recall (R)/ True Positive Rate: computes the fraction of positive examples that are correctly classified to the total number of positive examples evaluated.
  $$R=\frac{TP}{TP+FN}.$$
- Specificity (sp): indicates the fraction of negative examples that are correctly predicted.
  $$\mathrm{sp}=\frac{TN}{TN+FP}.$$
- Precision (P): measures the proportion of positive examples correctly predicted to the total number of positive predictions.
  $$p=\frac{TP}{TP+FP}.$$
- $F_{1}$ Score: is a measure of the harmonic mean of recall and precision. This is a good measure of performance when the classes are in-balanced.
  $$F_{1}\;\mathrm{Score}=\frac{2}{\frac{1}{R}+\frac{1}{P}}=2\left(\frac{RP}{R+P}\right).$$
- False Positive Rate (FPR): is the fraction of negative examples incorrectly classified to the total number of negative samples. It is also regarded as the complement of specificity.
  $$\mathrm{FPR}=\frac{FP}{FP+TN}=1-sp.$$

## 4. Results

This section presents the results of all experiments conducted in this work. Three CNN architectures Visual Geometric Group-19 (VGG-19), Densely Connected Convolutional Network-121 (DenseNet-121), and Deep Residual Network-50 (ResNet-50) were used to carry out experiments. The result of each model has some performance similarities as well as marked contrasts highlighted in the illustrations (loss and accuracy curves, confusion matrices and Receiver Operating Characteristic curves). With the goal of ML being finding a model that shows robust bias-variance trade-off, albeit, out of sample distribution (OOD) generalization. The hyperparameter choices and configurations in the present work where empirically-driven and are indicative of best practices in modeling with deep neural networks, reason being, deep network models are highly opaque and black-box in nature.

**Figure 9:**
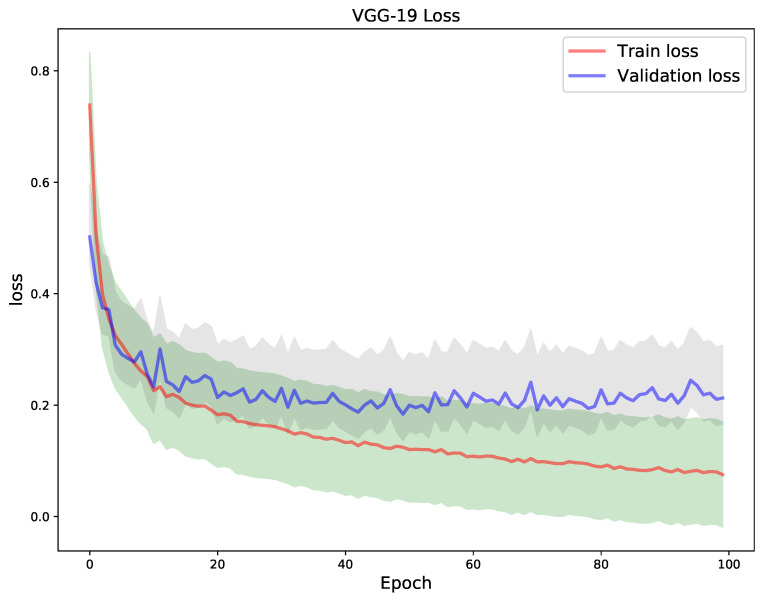
VGG-19 model was trained for 100 epochs. The plot depicts the train vs. validation loss.

**Figure 10:**
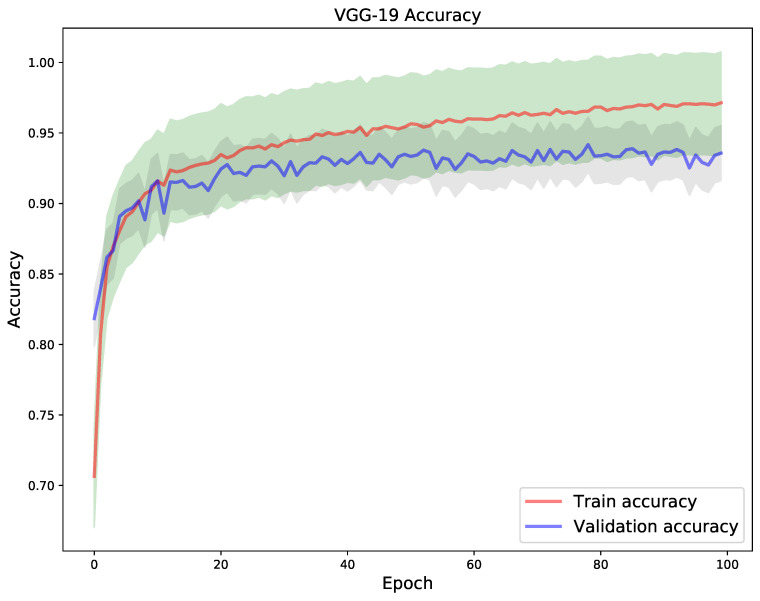
VGG-19 model was trained for 100 epochs. The plot on the right depicts the train vs. validation accuracy.

Using a 5-fold cross validation technique, Figure 9 shows the train and validation loss and Figure 10 shows the accuracy for train and validation for the VGG-19 model. With the aforementioned technique, the VGG-19 model obtained a best train and validation accuracy of 96.4% and 93.4% respectively, and a test accuracy of 93.99% superseding the other two models with respect to test accuracy. This is supported by the side-by-side comparison of the test image classification summary in Table 3.

**Figure 11:**
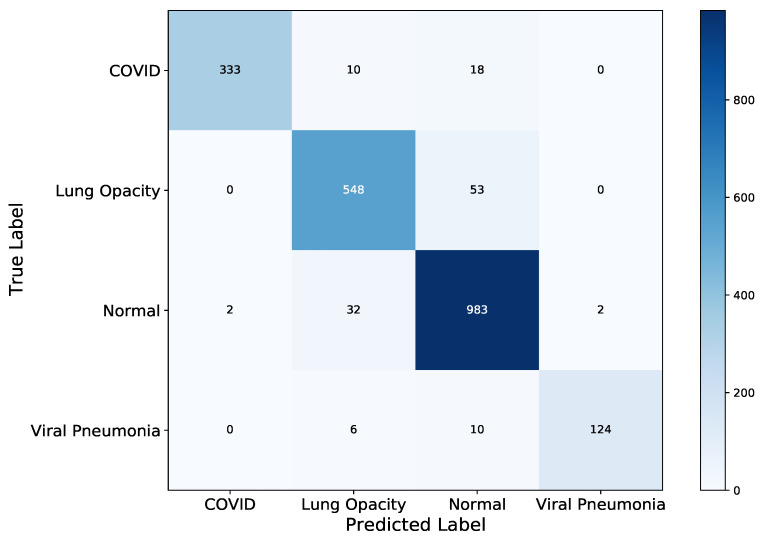
VGG-19 Test Confusion Matrix, where most X-ray images were classified correctly with few misclassifications for COVID-19 class.

**Figure 12:**
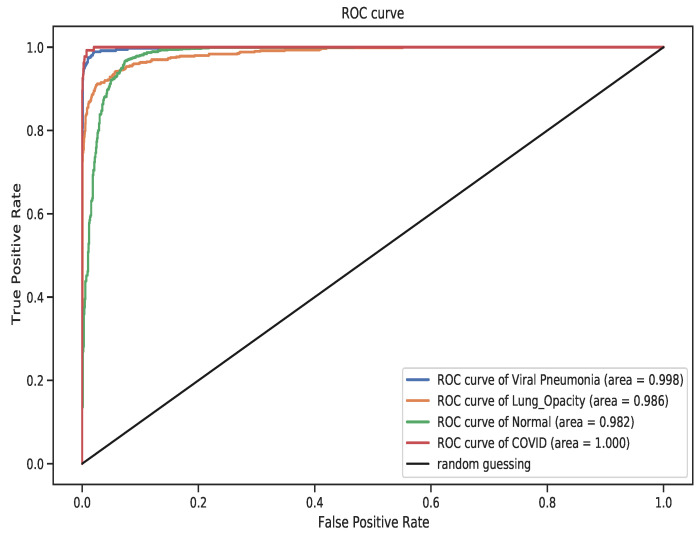
The Receiver Operator Characteristic Curve (ROC) where COVID-19 class obtained the high Area Under the Curve (AUC) of 1.0.

However, the accuracy is not very informative in situations where a dataset is highly in-balanced as in the current work. Secondly, the objective of developing a model highly influences the performance metric choice to be made. Thus, there is a trade-off where a model should have a high sensitivity towards a certain class. In the current context, COVID-19 which if incorrectly classified albeit goes on detected can lead to a massive infection rate in the community retarding the containment of the virus efforts. So, the confusion matrix in Figure 11 and the Receiver Operator Characteristic curve in Figure 12 depict the fine-grain performance of the VGG-19 model on each XCR image class. The model generally, performed well across the four classes with Viral Pneumonia having the least misclassified examples relative to the class size. The ability of VGG-19 to disentangle and correctly classified these Viral Pneumonia images is largely due to its distinct Gaussian-like class distribution as shown in Figure 8 whilst Normal and Lung Opacity have similar class distributions leading to problems of correctly disentangling and classifying all examples from both classes correctly. This similarity between the Normal and Lung Opacity class distributions has consistently led to high misclassification of examples between the two classes across all trained models.

To further gain a deeper understanding of the classifiers performance across the four classes, the above ROC curve in Figure 12, characterizes the AUC. The AUC gives an indicator of the classifiers performance per class across the test dataset. And from the ROC curve, the VGG-19 model showed a remarkable AUC score of 1.0 across the COVID-19 images. The advantage of this measure is, it contrasts the True Positive Rate to the False Positive Rate. So a higher AUC indicates a class-level performance of the model and the reverse holds true. In summary, the model was able to learn disentangled representations of COVID-19 images and classify them better than the three other classes.

**Figure 13:**
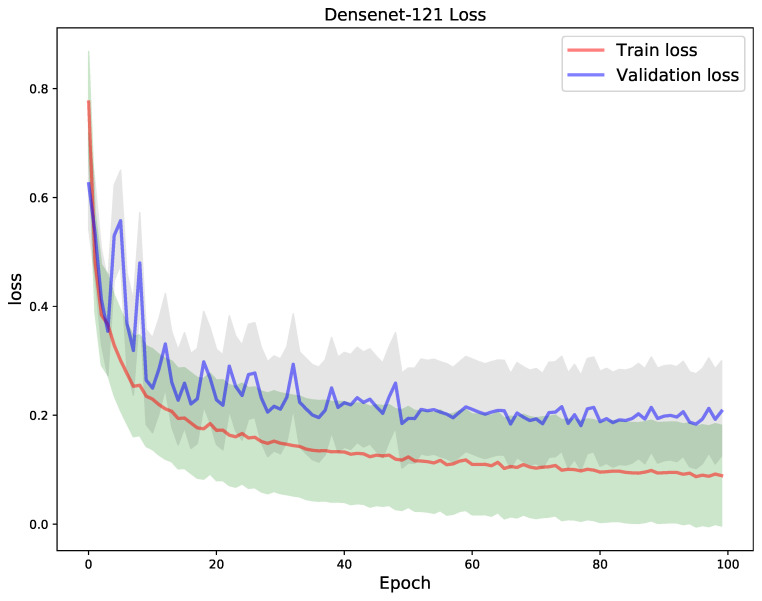
A DenseNet-121 model trained during 100 epochs: train vs. validation loss.

**Figure 14:**
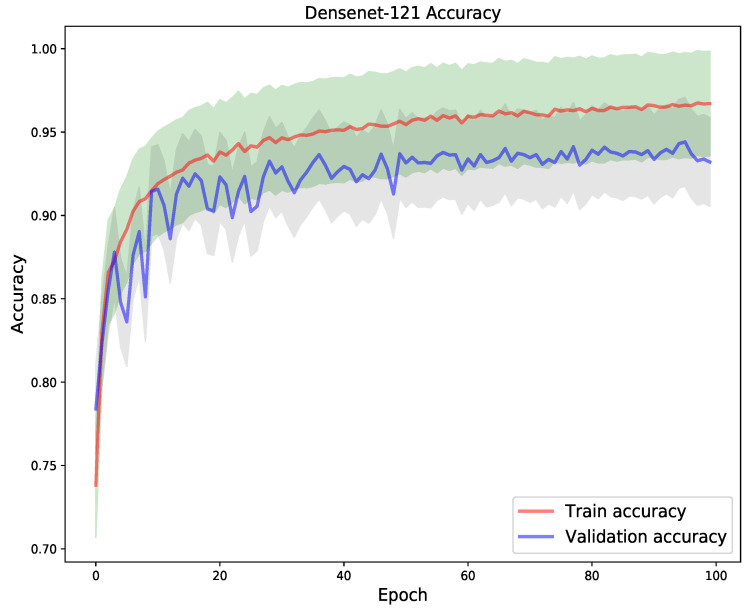
Train vs validation accuracy. The model showed unstable convergence behaviour in the first 45 epochs as the graphs show off-shooting effects in both train and loss metrics.

In the next experiment, a DenseNet-121 model was trained for 100 epochs, using the same test size used to testing the VGG-19 model. The goal of the experiment, was to compare the models generalization performance so as to perform a selection of the best model. From Figure 13 and Figure 14, one can notice that the model had high degree of unstable convergence to the optima, i.e., in both the train and validation loss as well as the train and validation accuracy graph. The train loss remained consistently lower than the validation, though weight decaying, class weighting, learning rate scheduling, checkpoints and regularization techniques we used. A similar phenomenon was noticed with the train and validation accuracy curves. However, the DenseNet-121 model shows a greater degree of convergence stability in the last 50 epochs as opposed to the VGG-19 model. This is indicative of a lower spread (upper and lower standard deviations) of the curves. Nonetheless, the model achieved an overall train, validation and test accuracy of 97.4%, 93.58% and 93.24%, respectively.

**Figure 15:**
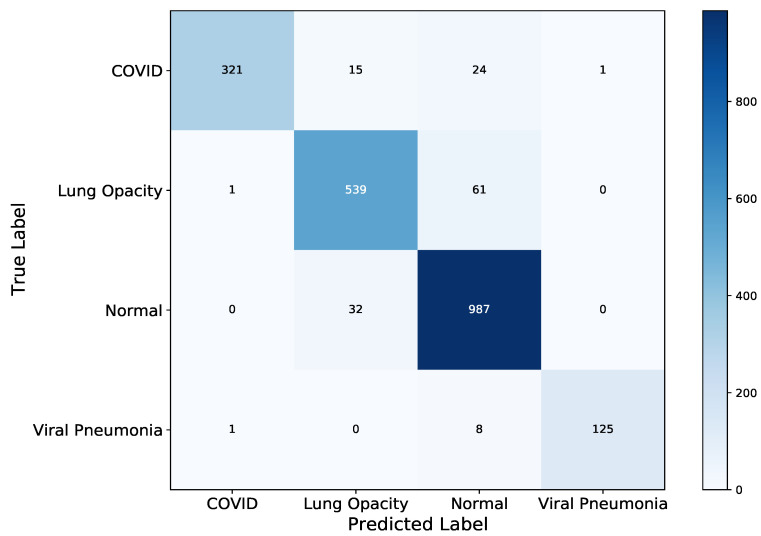
DenseNet-121 Test Confusion Matrix, most of the test XCR images were classified correctly; however, Lung Opacity, and Normal classes have the highest total misclassification of 61 and 32, respectively. DenseNet-121 has a higher misclassification total than VGG-19.

**Figure 16:**
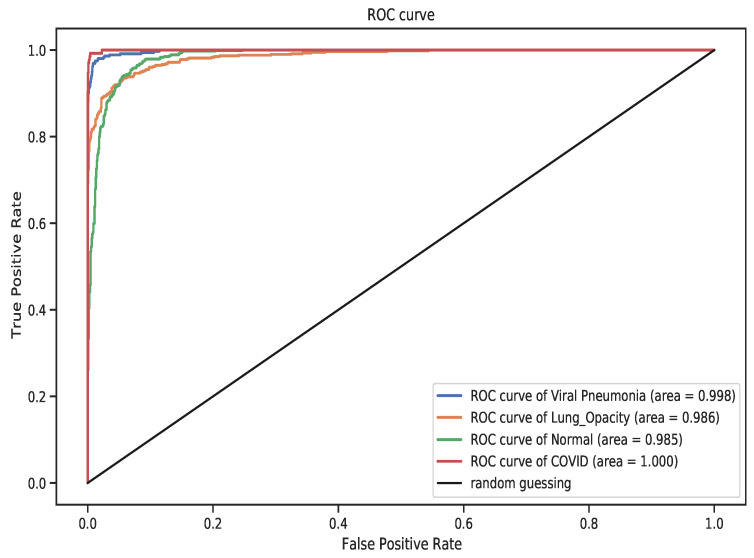
The ROC curve. COVID-19 achieved the highest AUC of 1.0 followed by Viral Pneumonia class having 0.998 whilst the two remaining classes have AUC of 0.98.

To better understand and analyze the class-level performance of the model, Figure 15 and Figure 16 show the test confusion matrix and the ROC curve respectively. From the confusion matrix, most of the images were classified correctly; however, the same problem of high misclassification for Normal and Lung Opacity classes persists. This clearly warrants further inquire into the data generation process (at least for the classes in question). It is worth noting that, the dataset was aggregated from multiple sources, which can, inevitably introduce a range of problems from measurement errors, to wrong class labelling. Given, that the field of radiology extensively requires dedicated training and specialization, annotating medical images relies on domain knowledge and tracking problems is painstakingly difficult. Nonetheless, the four classes showed a remarkable AUC performance, relative the to other two models. COVID-19 class shows a consistent 1.0 AUC score in the DenseNet-121 model while Lung Opacity has the least AUC score of 0.982.

**Figure 17:**
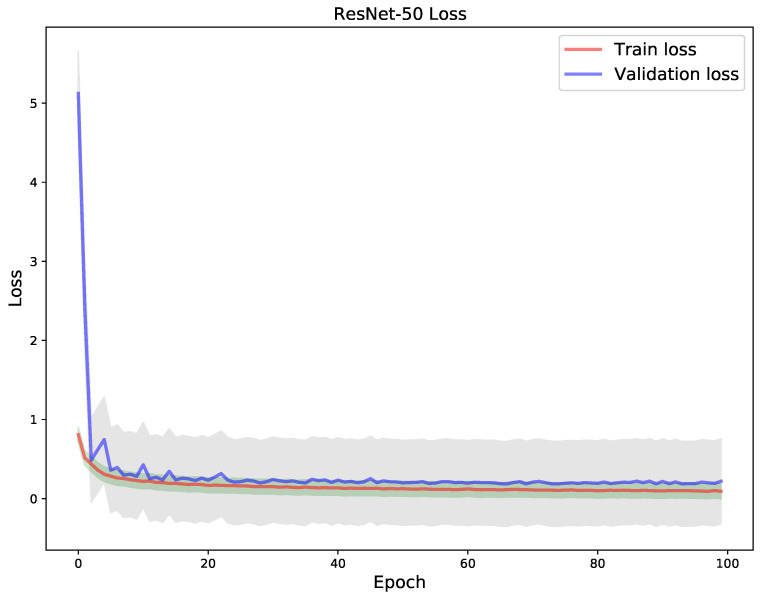
Train vs validation loss of ResNet-50.

**Figure 18:**
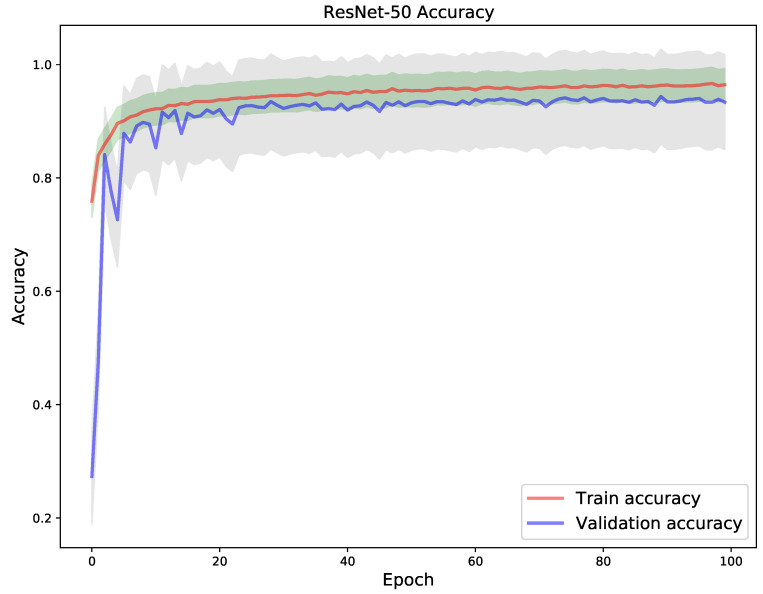
Train vs validation accuracy of ResNet-50.

Finally, a ResNet-50 pre-trained CNN model was used for transfer learning in the XCR dataset. The model after fine-tuning obtained an overall test accuracy of 0.938534. One can discern from the loss in Figure 17 and accuracy Figure 18 that the model had a nearly smooth convergence to the optimum during training. This is indicative by the perfect fit relationship between the train and validation loss and accuracy curves across all 100 epochs. This phenomenon can be explained by the robust representational power of ResNet-50 in tackling the vanishing gradient problem through residual connections in its architectural formulation. The residual connection in ResNet, allows not only the building of deep representational stacks of hidden layers but the preservation of information and gradient flow in layer-wise transformations in a CNN network.

**Figure 19:**
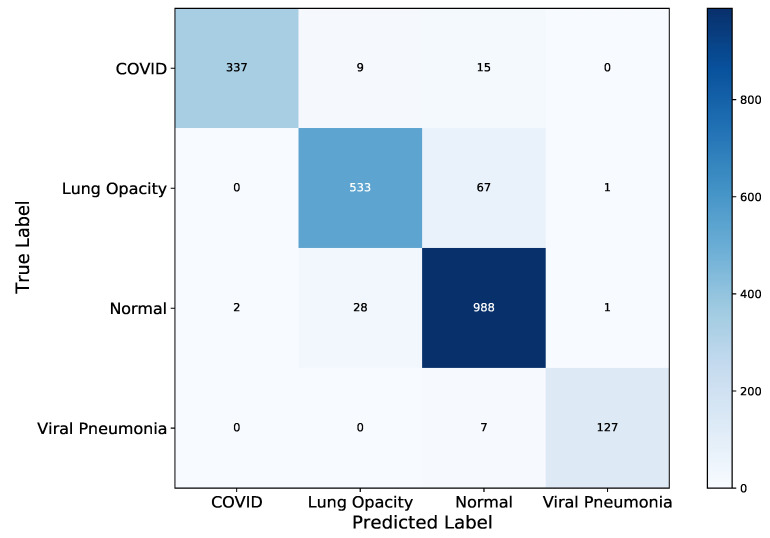
ResNet-50 Confusion Matrix on the left graph. Like the other two models, the Lung Opacity and Normal XCR image classes showed the highest two misclassification.

**Figure 20:**
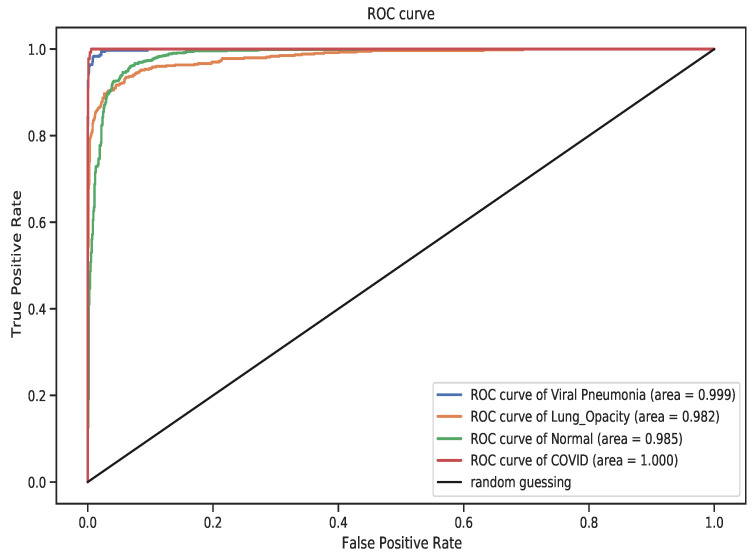
The ROC curve for ResNet-50 model. The overall performance of the model for the four classes was excellent with COVID-19 class obtaining AUC of 1.0.

The test confusion matrix is shown in Figure 19 to comparatively assess class-level performance of the model. In this figure, as indicated in the former models, Lung Opacity has the highest misclassification with a total of 68 images followed by the normal class having 31 misclassified images. A subset of images in these two classes appear to be entangled, thus one being predicted as the other by the model. However, ResNet-50 has the least number of misclassification with a total of 140 followed by VGG-19 having a total of 137 misclassified images and DenseNet-121 with 153 misclassified XCR images. We showed the ROC curve in Figure 20 that depicts the False Positive Rate (FPR) against the True Positive Rate (TPR) for the four classes under study. The results of the experiments indicate that ResNet-50 achieved a better AUC performance in contrast to VGG-19 and DenseNet-121 models. Based on the analysis of the performance of the three models, we observe that ResNet-50 is well suited for the detection and classification of traditional pneumonia and pneumonia induced by the COVID-19 from Chest X-ray images.

**Figure 21:**
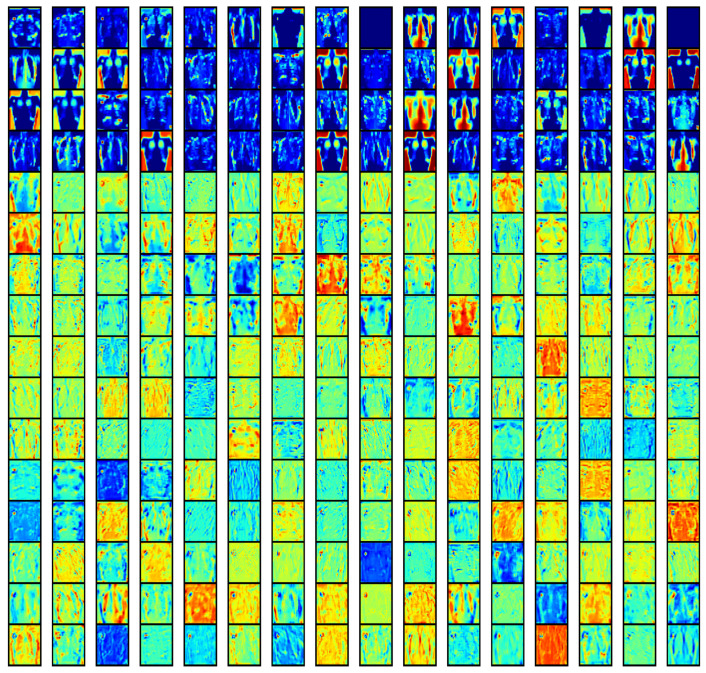
In this diagram (ResNet-50 convolution layer 48 with 256 filter activation maps in a 16 × 16 grid), we see the emergence of XCR image structure in the feature maps after Transfer Learning. This shows that the representation of the network has learnt inherent invariant regularities in the XCR dataset.

To understand the nature of the learnt representation in the fine-tuned ResNet-50 model, the activation map for layer 48 is shown in Figure 21. In this layer, the model learned the latent factors of variation in the chest X-ray images as shown by the first four rows in the 16 × 16 grid of filters. This indication of specific firing patterns of the filters for separate image classes is a striking feature of hierarchy representations of disentangled representations learnt in the CNN model.

## 5. Discussion

The maturation of machine learning and computer vision fields have offset remarkable research interest in their application to medical image analysis. Traditionally, medical image analysis of patients is done by radiologist. This process is laborious and time-consuming. Thus extensive scholarly work has been done in medical image analysis with computer vision and machine learning to help improve healthcare outcomes. The outbreak of the COVID-19 caused by the Severe Acute Respiratory Syndrome Coronavirus 2 (SARS-CoV-2) has led to extensive research into the application of Deep Learning for fast and accurate detection of the disease. Deep Learning for the detection of COVID-19 from chest X-ray modality has primary been used in prior work as a viable complementary test method to the Reverse Transcriptase-Polymerase Chain Reaction (RT–PCR). The existing literature on deep learning models for the detection of COVID-19 mostly utilize unrealistic experimental setups (small, overly handcrafted and augmented datasets, performance scoring on validation as opposed to test set and an ensemble of models that compound compute cost thus introducing reproducibility inequity). On that ground, we carried out experiment using a noisy dataset of chest X-ray images collected from 20 health centres across the world. This was done primary to tackle the problems of distribution shift and concept drift. Distribution shift (co-variate shift) happens where the distribution of independent variables shift potentially due to spatio-temporal variability in latent processes. While concept drift occurs in the change of the statistical properties of target variables over time in unforeseen ways. A survey of recent work in detecting and classifying COVID-19 from chest X-ray images is presented in Table 1. Our result shows an overall accuracy of 94.0% using a VGG-19 model. However, upon fine-grain performance analysis, we showed that ResNet-50 has the best test performance with respect to ROC curve analysis.

## 6. Conclusions and Future Directions

In this present work, we proposed a framework for the diagnosis and classification of traditional pneumonia and pneumonia induced by the COVID-19 from Chest X-ray images. We demonstrated that Transfer Learning shows a promising direction of training medical diagnostic deep learning models where access to annotated dataset is limited as manual labelling in such setting is very laborious and expensive because it requires domain expertise. Thus, using 5-fold cross validation, our work indicates the potential utilization of transfer learning to aid fast and accurate early detection of COVID-19 especially in asymptomatic patients. Nonetheless, further inquiry is required to use hyperparameter optimization techniques such as Grid Search, Bayesian Optimization or Evolutionary Optimization, to find the right set of hyperparameters for better test performance improvement. This approach was a limitation in our study because of limited compute power to carryout such hyperparameter space search in a deep learning setting. Additionally, we compared our work with the literature related to the detection of COVID-19 on chest X-ray images. Future research may compare computed tomography modality and chest X-ray to determine which one is well suited for diagnosing the COVID-19.

## Figures and Tables

**Table 1:** Presents a survey of proposed COVID-19 chest X-ray classification and/or detection methodology, the dataset(s) used in the research, and reported accuracy for the proposed approach.

Reference	Number of Images	Classes and Size	Method(s)	Reported Accuracy
Khan et al. [37]	1251	Normal (310) Pneumonia Bacterial (330) Pneumonia Virus (327) COVID-19 (284)	CNN (ConNet)	89%
Ozturk et al. [46]	1625	Pneumonia (500) No Findings (1000) COVID-19 (125)	Dark CovidNet	87.02%
Apostolopoulos and Mpesiana [32]	1427 1442	Dataset (i) Pneumonia Bacteria (700) Normal (504) COVID-19 (224) Dataset (ii) Pneumonia Bacteria and Viral (714) Normal (504) COVID-19 (224)	CNN, MobileNet v2	96.78%
Wang et al. [10]	13975	Pneumonia (5538) Normal (8066) COVID-19 (358)	DNN, VGG-19, ResNet-50, CovidNet	93.3%
Sethy and Behera [47]	381	Pneumonia (127) Normal (127) COVID-19 (127)	CNN, ResNet-50 + SVM	95.35%
El-Din Hemdan et al. [33]	50	Normal (25) COVID-19 (25)	D-CNN, VGG-19, DenseNet-201	98.0%
Keles et al. [48]	810	Normal (350) COVID-19 (210) Viral Pneumonia (350)	CNN, Covid-ResNet, Covid-CNNet	97.6%
Narayanan et al. [49]	5856	Normal (1583) Viral Pneumonia (1493) Bacterial Pneumonia (2780)	ResNet-50, DenseNet-201, Inception-v3, Xception	98.0%
Ghoshal and Tucker [50]	5941	Normal (1583) Viral Pneumonia (1504) Bacterial Pneumonia (2786) COVID-19 (1968)	CNN, Bayesian ResNet-50 v2	89.92%
Chowdhury et al. [22]	3487	Normal (1579) Viral Pneumonia (1485) COVID-19 (423)	DCNN, CheXNet + DenseNet-201	99.7%
Zhang et al. [51]	1531	Pneumonia (1431) COVID-19 (100)	CNN, Classification Grad-CAM	95.13% AUC
Abbas et al. [31]	195	Normal (80) COVID-19 (105)	D-CNN, DeTraC	95.12%
Karim et al. [52]	16,995	Normal (8066) Pneumonia (8614) COVID-19 (259)	DNN, Deep Covid Explainer	PPV 96.12%
Sitaula and Hossain [53]	1125 1638	Dataset (i) No Findings (N/A) Pneumonia (N/A) COVID-19 (N/A) Dataset (ii) Normal (310) Pneumonia Bacteria (330) COVID-19 (327) Viral Pneumonia (327)	VGG-16, VGG-19	87.49%
Pham [54]	1124	Dataset (i) Normal (721) COVID-19 (403) Dataset (ii) Normal (438) COVID-19 (438) Dataset (iii) Normal (876) COVID-19 (438) Viral Pneumonia (436)	CNN, AlexNet, GoogleNet, SqueezeNet	99.0%
Chandra et al. [38]	542 80 680	Dataset (i) Normal (19) COVID-19 (434) Pneumonia (89) Dataset (ii) Normal (80) Dataset (iii) Normal (345) Pneumonia (345)	KNN, ANN, DT, SVM	93.41%

**Table 2:** Presents the four concept classes which comprise COVID-19, Normal, Lung Opacity, and Viral Pneumonia. The normal class has the majority samples followed by Lung Opacity which is almost twice the former. In addition, the COVID-19 class has the third-largest class size followed by the Viral Pneumonia class. The table further gives a detailed indication of the train, validation, and test splits for each class.

Set	COVID-19	Normal	Lung Opacity	Viral Pneumonia
Train	2604	7339	4329	969
Validation	651	1834	1082	242
Test	361	1019	601	134
Total	3616	10,192	6012	1345

**Table 3:** A summary of total images classified correctly and incorrectly by VGG-19, DenseNet-121, and ResNet-50 using a total test dataset of 2115 images. Amongst the three models, VGG-19 demonstrated high accuracy of XCR image classification with only 127 misclassifications.

Model	Correct Classification	Incorrect Classification
VGG-19	1988	127
DenseNet-121	1972	143
ResNet-50	1985	130

## Data Availability

Publicly available datasets were analyzed in this study. This data can be found here: https://www.kaggle.com/tawsifurrahman/covid19-radiography-database (accessed on 5 August 2021).

## References

[1] Ciotti M., Ciccozzi M., Terrinoni A., Jiang W.C., Wang C.B., Bernardini S. (2020). The COVID-19 pandemic. Crit. Rev. Clin. Lab. Sci..

[2] Chan J.F.W., Yuan S., Kok K.H., To K.K.W., Chu H., Yang J., Xing F., Liu J., Yip C.C.Y., Poon R.W.S. (2020). A familial cluster of pneumonia associated with the 2019 novel coronavirus indicating person-to-person transmission: A study of a family cluster. Lancet.

[3] World Health Organization (2020). Coronavirus Disease 2019 (COVID-19), Situation Report.

[4] Ahsan M., Based M., Haider J., Kowalski M. (2021). COVID-19 Detection from Chest X-ray Images Using Feature Fusion and Deep Learning. Sensors.

[5] Ledford H., Cyranoski D., Van Noorden R. (2020). The UK has approved a COVID vaccine-here’s what scientists now want to know. Nature.

[6] Li Y., Tenchov R., Smoot J., Liu C., Watkins S., Zhou Q. (2021). A comprehensive review of the global efforts on COVID-19 vaccine development. ACS Cent. Sci..

[7] Kim J.H., Marks F., Clemens J.D. (2021). Looking beyond COVID-19 vaccine phase 3 trials. Nat. Med..

[8] Logunov D.Y., Dolzhikova I.V., Shcheblyakov D.V., Tukhvatulin A.I., Zubkova O.V., Dzharullaeva A.S., Kovyrshina A.V., Lubenets N.L., Grousova D.M., Erokhova A.S. (2021). Safety and efficacy of an rAd26 and rAd5 vector-based heterologous prime-boost COVID-19 vaccine: An interim analysis of a randomised controlled phase 3 trial in Russia. Lancet.

[9] Chen Z., Zhang L. (2021). Meet the Challenges of Mass Vaccination against COVID-19. Explor. Res. Hypothesis Med..

[10] Wang J., Peng Y., Xu H., Cui Z., Williams R.O. (2020). The COVID-19 vaccine race: Challenges and opportunities in vaccine formulation. AAPS PharmSciTech.

[11] Forni G., Mantovani A. (2021). COVID-19 vaccines: Where we stand and challenges ahead. Cell Death Differ..

[12] Binnicker M.J. (2020). Challenges and Controversies to Testing for COVID-19. J. Clin. Microbiol..

[13] Tavare A.N., Braddy A., Brill S., Jarvis H., Sivaramakrishnan A., Barnett J., Creer D.D., Hare S.S. (2020). Managing high clinical suspicion COVID-19 inpatients with negative RT-PCR: A pragmatic and limited role for thoracic CT. Thorax.

[14] Wang L., Lin Z.Q., Wong A. (2020). Covid-net: A tailored deep convolutional neural network design for detection of covid-19 cases from chest X-ray images. Sci. Rep..

[15] Afzal A. (2020). Molecular diagnostic technologies for COVID-19: Limitations and challenges. J. Adv. Res..

[16] World Health Organization (2020). Use of Chest Imaging in COVID-19: A Rapid Advice Guide, 11 June 2020.

[17] Kong W., Agarwal P.P. (2020). Chest imaging appearance of COVID-19 infection. Radiol. Cardiothorac. Imaging.

[18] Davies H., Wathen C., Gleeson F. (2011). The risks of radiation exposure related to diagnostic imaging and how to minimise them. Bmj.

[19] Cherian T., Mulholland E.K., Carlin J.B., Ostensen H., Amin R., Campo M.d., Greenberg D., Lagos R., Lucero M., Madhi S.A. (2005). Standardized interpretation of paediatric chest radiographs for the diagnosis of pneumonia in epidemiological studies. Bull. World Health Organ..

[20] Franquet T. (2001). Imaging of pneumonia: Trends and algorithms. Eur. Respir. J..

[21] Ng M.Y., Lee E.Y., Yang J., Yang F., Li X., Wang H., Lui M.M.s., Lo C.S.Y., Leung B., Khong P.L. (2020). Imaging profile of the COVID-19 infection: Radiologic findings and literature review. Radiol. Cardiothorac. Imaging.

[22] Chowdhury M.E.H., Rahman T., Khandakar A., Mazhar R., Kadir M.A., Mahbub Z.B., Islam K.R., Khan M.S., Iqbal A., Emadi N.A. (2020). Can AI Help in Screening Viral and COVID-19 Pneumonia?. IEEE Access.

[23] Baltruschat I., Nickisch H., Grass M., Knopp T., Saalbach A. (2019). Comparison of Deep Learning Approaches for Multi-Label Chest X-ray Classification.

[24] Siddiqi R. Automated pneumonia diagnosis using a customized sequential convolutional neural network. Proceedings of the 2019 3rd International Conference on Deep Learning Technologies.

[25] Ebiele J., Ansah-Narh T., Djiokap S., Proven-Adzri E., Atemkeng M. Conventional Machine Learning based on Feature Engineering for Detecting Pneumonia from Chest X-rays. Proceedings of the 2020 ACM Conference of the South African Institute of Computer Scientists and Information Technologists.

[26] Kikkisetti S., Zhu J., Shen B., Li H., Duong T. (2020). Deep-learning convolutional neural networks with transfer learning accurately classify COVID-19 lung infection on portable chest radiographs. PeerJ.

[27] Ardabili S.F., Mosavi A., Ghamisi P., Ferdinand F., Varkonyi-Koczy A.R., Reuter U., Rabczuk T., Atkinson P.M. (2020). COVID-19 Outbreak Prediction with Machine Learning. Algorithms.

[28] Pinter G., Felde I., Mosavi A., Ghamisi P., Gloaguen R. (2020). COVID-19 Pandemic Prediction for Hungary; A Hybrid Machine Learning Approach. Mathematics.

[29] Burke R.M., Shah M.P., Wikswo M.E., Barclay L., Kambhampati A., Marsh Z., Cannon J.L., Parashar U.D., Vinjé J., Hall A.J. (2019). The norovirus epidemiologic triad: Predictors of severe outcomes in US norovirus outbreaks, 2009–2016. J. Infect. Dis..

[30] Ahammed K., Satu M.S., Abedin M.Z., Rahaman M.A., Islam S.M.S. (2020). Early Detection of Coronavirus Cases Using Chest X-ray Images Employing Machine Learning and Deep Learning Approaches. medRxiv.

[31] Abbas A., Abdelsamea M.M., Gaber M.M. (2021). Classification of COVID-19 in chest X-ray images using DeTraC deep convolutional neural network. Appl. Intell..

[32] Apostolopoulos I.D., Mpesiana T.A. (2020). Covid-19: Automatic detection from X-ray images utilizing transfer learning with convolutional neural networks. Phys. Eng. Sci. Med..

[33] El-Din Hemdan E., Shouman M.A., Karar M.E. (2020). Covidx-net: A framework of deep learning classifiers to diagnose covid-19 in X-ray images. arXiv.

[34] Karar M.E., Hemdan E.E.D., Shouman M.A. (2021). Cascaded deep learning classifiers for computer-aided diagnosis of COVID-19 and pneumonia diseases in X-ray scans. Complex Intell. Syst..

[35] Minaee S., Kafieh R., Sonka M., Yazdani S., Soufi G.J. (2020). Deep-covid: Predicting covid-19 from chest X-ray images using deep transfer learning. Med. Image Anal..

[36] Heidari M., Mirniaharikandehei S., Khuzani A.Z., Danala G., Qiu Y., Zheng B. (2020). Improving the performance of CNN to predict the likelihood of COVID-19 using chest X-ray images with preprocessing algorithms. Int. J. Med. Inform..

[37] Khan A.I., Shah J.L., Bhat M.M. (2020). CoroNet: A deep neural network for detection and diagnosis of COVID-19 from chest X-ray images. Comput. Methods Programs Biomed..

[38] Chandra T.B., Verma K., Singh B.K., Jain D., Netam S.S. (2021). Coronavirus disease (COVID-19) detection in Chest X-Ray images using majority voting based classifier ensemble. Expert Syst. Appl..

[39] Ismael A.M., Şengür A. (2021). Deep learning approaches for COVID-19 detection based on chest X-ray images. Expert Syst. Appl..

[40] Karthik R., Menaka R., Hariharan M. (2021). Learning distinctive filters for COVID-19 detection from chest X-ray using shuffled residual CNN. Appl. Soft Comput..

[41] Ohata E.F., Bezerra G.M., das Chagas J.V.S., Neto A.V.L., Albuquerque A.B., de Albuquerque V.H.C., Reboucas Filho P.P. (2020). Automatic detection of COVID-19 infection using chest X-ray images through transfer learning. IEEE/CAA J. Autom. Sin..

[42] De Moura J., García L.R., Vidal P.F.L., Cruz M., López L.A., Lopez E.C., Novo J., Ortega M. (2020). Deep convolutional approaches for the analysis of covid-19 using chest X-ray images from portable devices. IEEE Access.

[43] Duran-Lopez L., Dominguez-Morales J.P., Corral-Jaime J., Vicente-Diaz S., Linares-Barranco A. (2020). COVID-XNet: A custom deep learning system to diagnose and locate COVID-19 in chest X-ray images. Appl. Sci..

[44] Shorfuzzaman M., Hossain M.S. (2021). MetaCOVID: A Siamese neural network framework with contrastive loss for n-shot diagnosis of COVID-19 patients. Pattern Recognit..

[45] Shankar K., Perumal E. (2020). A novel hand-crafted with deep learning features based fusion model for COVID-19 diagnosis and classification using chest X-ray images. Complex Intell. Syst..

[46] Ozturk T., Talo M., Yildirim E.A., Baloglu U.B., Yildirim O., Acharya U.R. (2020). Automated detection of COVID-19 cases using deep neural networks with X-ray images. Comput. Biol. Med..

[47] Sethy P.K., Behera S.K. (2020). Detection of coronavirus disease (covid-19) based on deep features. Preprints.

[48] Keles A., Keles M.B., Keles A. (2021). COV19-CNNet and COV19-ResNet: Diagnostic inference Engines for early detection of COVID-19. Cogn. Comput..

[49] Narayanan B.N., Hardie R.C., Krishnaraja V., Karam C., Davuluru V.S.P. (2020). Transfer-to-transfer learning approach for computer aided detection of COVID-19 in chest radiographs. AI.

[50] Ghoshal B., Tucker A. (2020). Estimating uncertainty and interpretability in deep learning for coronavirus (COVID-19) detection. arXiv.

[51] Zhang J., Xie Y., Li Y., Shen C., Xia Y. (2020). Covid-19 screening on chest X-ray images using deep learning based anomaly detection. arXiv.

[52] Karim M., Döhmen T., Rebholz-Schuhmann D., Decker S., Cochez M., Beyan O. (2020). Deepcovidexplainer: Explainable covid-19 predictions based on chest X-ray images. arXiv.

[53] Sitaula C., Hossain M.B. (2021). Attention-based VGG-16 model for COVID-19 chest X-ray image classification. Appl. Intell..

[54] Pham T.D. (2021). Classification of COVID-19 chest X-rays with deep learning: New models or fine tuning?. Health Inf. Sci. Syst..

[55] Krizhevsky A., Sutskever I., Hinton G.E. (2012). Imagenet classification with deep convolutional neural networks. Adv. Neural Inf. Process. Syst..

[56] Simonyan K., Zisserman A. (2014). Very deep convolutional networks for large-scale image recognition. arXiv.

[57] He K., Zhang X., Ren S., Sun J. Deep residual learning for image recognition. Proceedings of the IEEE conference on computer vision and pattern recognition.

[58] Szegedy C., Liu W., Jia Y., Sermanet P., Reed S., Anguelov D., Erhan D., Vanhoucke V., Rabinovich A. Going deeper with convolutions. Proceedings of the IEEE conference on computer vision and pattern recognition.

[59] Dosovitskiy A., Beyer L., Kolesnikov A., Weissenborn D., Zhai X., Unterthiner T., Dehghani M., Minderer M., Heigold G., Gelly S. (2020). An image is worth 16 × 16 words: Transformers for image recognition at scale. arXiv.

[60] Hubel D.H., Wiesel T.N. (1962). Receptive fields, binocular interaction and functional architecture in the cat’s visual cortex. J. Physiol..

[61] Marr D. Representing Visual Information. https://apps.dtic.mil/sti/citations/ADA055045.

[62] Vayá M.d.l.I., Saborit J.M., Montell J.A., Pertusa A., Bustos A., Cazorla M., Galant J., Barber X., Orozco-Beltrán D., García-García F. (2020). Bimcv covid-19+: A large annotated dataset of rx and ct images from covid-19 patients. arXiv.

[63] Cohen J.P., Morrison P., Dao L., Roth K., Duong T.Q., Ghassemi M. (2020). COVID-19 Image Data Collection: Prospective Predictions Are the Future. arXiv.

[64] Haghanifar A., Majdabadi M.M., Choi Y., Deivalakshmi S., Ko S. (2020). COVID-CXNet: Detecting COVID-19 in Frontal Chest X-ray Images using Deep Learning. arXiv.

[65] Kermany D.S., Goldbaum M., Cai W., Valentim C.C., Liang H., Baxter S.L., McKeown A., Yang G., Wu X., Yan F. (2018). Identifying medical diagnoses and treatable diseases by image-based deep learning. Cell.

[66] Wang N., Liu H., Xu C. Deep learning for the detection of COVID-19 using transfer learning and model integration. Proceedings of the 2020 IEEE 10th International Conference on Electronics Information and Emergency Communication (ICEIEC).

[67] Benbrahim H., Hachimi H., Amine A. (2020). Deep transfer learning with apache spark to detect covid-19 in chest X-ray images. Rom. J. Inf. Sci. Technol..

